# Spectrum of actionable genetic variants in acute myeloid leukaemia patients from Pakistan: a study based on next-generation sequencing

**DOI:** 10.3332/ecancer.2026.2132

**Published:** 2026-05-27

**Authors:** Sana Khurram, Ali Abdul Basit, Muhammad Asif Qureshi

**Affiliations:** 1 Department of Pathology, Dow University of Health Sciences, Karachi 74200, Pakistan; 2 Rawalpindi Medical University, Rawalpindi 46000, Pakistan; a https://orcid.org/0009-0001-3739-415X; b https://orcid.org/0009-0003-1624-208X; c https://orcid.org/0000-0002-8370-1069

**Keywords:** aute myeloid leukaemia, next-generation sequencing, genetic variation, Pakistan, mutation, hematologic neoplasms, molecular targeted therapy

## Abstract

Acute myeloid leukaemia (AML) is a genetically diverse malignancy with a high incidence in Pakistan. But patients rarely benefit from personalized care due to the unavailability of molecular diagnostics. This study aims to identify actionable genetic variants in adult Pakistani AML patients by using the inDxTM Extended AML/myelodysplastic syndrome panel for targeted next generation sequencing (NGS). Nine patients were included (median age = 42 years, 77.8% males). Mutations were found in six (66.7%) patients. The total number of mutations was 13 and these mutated 11 different genes, nine genes being affected in single cases (7.7%) and two genes: WT1 and DNMT3A, were mutated in two cases (15.4% each). Missense mutations constituted 69.2% of all 13 mutations. The variant allele frequencies for these mutations ranged from 7% to 54%. A number of clinically important mutations were observed, including an adverse WT1 variant, a favourable NPM1 mutation, targetable IDH1/JAK2 dual mutations and a myelodysplasia-related U2AF1 mutation. Co-mutation of DNMT3A with KRAS/NRAS or NPM1 indicated complex clonal dynamics. One of the three patients with no detectable mutations on NGS, was found Breakpoint cluster region-abelson murine leukaemia viral oncogene homolog 1-positive through polymerase chain reaction. This highlights the limitations of NGS panel, and advocates for the need for a multi-layered diagnostic approach. These findings support the viability of NGS in detecting clinically significant mutations in low-resource settings to guide risk stratification and personalized treatment planning. This study highlights the need for broader NGS integration in routine care and larger regional studies to improve AML management in Pakistan.

## Introduction

Acute myeloid leukaemia (AML) is a genetically heterogeneous clonal malignancy of myeloid precursors [1] and is the most common acute leukaemia in adults [2]. These tumours comprise approximately 80% of adult leukaemia cases [3]. Globally, the incidence and mortality of AML have been increasing, with particularly high rates observed in South Asia [4]. Despite recent advances in diagnostic and therapeutic modalities, AML continues to carry a poor prognosis, especially in elderly patients and those with high-risk genetic features [5,6]. It is therefore highly relevant to investigate molecular players associated with AML pathogenesis in order to identify novel molecules of diagnostic, therapeutic and prognostic significance in these patients.

In Pakistan, AML is the most frequently diagnosed leukaemia, comprising 35.9% of all diagnosed leukaemia cases [7,8], with a leukaemia incidence of 4.3 per 100,000, even higher than those reported for the United States highlighted by the GLOBOCAN 2020 [9]. Notably, and in contrast to the rest of the world, AML tends to present at a much younger age (mean age of ~36.7 years) in Pakistani populations, with a male predominance (M:F = 1.4:1) [7,8].

While cytogenetic/molecular studies from Pakistan in the context of AML patients are scarce, it has been reported that roughly 28.3% of AML cases from Pakistan harbor some degree of chromosomal abnormalities [10]. Favourable-risk abnormalities such as t(8;21), inv(16) and t(15;17) are reported, while complex karyotypes and monosomy 7 are associated with adverse prognosis [11]. Additionally, mutations including *NPM1*, *FLT3-ITD* [12], *MLL::AF9* [13] and *RUNX1-RUNX1T1* [10] have been reported, with *FLT3-ITD* prevalence ranging from 17.3% to 22% and D835 mutations in 6.3% of cases [14,15]. Despite these data, routine molecular profiling using next-generation sequencing (NGS) remains limited in Pakistan and are yet not part of routine AML patient workup. Moreover, AML-specific survival data from Pakistan are feeble.

The use of NGS worldwide has undoubtedly transformed the molecular diagnostic/therapeutic/prognostic landscape of AML patients. Additionally, NGS has been essential in developing treatment plans for hematological malignancies, particularly AML. However, a major challenge to its application in standard clinical practice is its high cost. As a result, it is only applied in situations where there is strong clinical evidence in limited-resource settings, such as Pakistan. Nevertheless, NGS is still a valuable tool that offers precise diagnosis, risk assessment and selection of treatment therapy.

It is noteworthy that the International Consensus Classification (ICC) and European Leukaemia Net (ELN) 2022 recommend using genetic mutations in biomarkers such as NPM1, FLT3, TP53 and myelodysplasia-related (MR) mutations such as ASXL1, RUNX1, EZH2, SRSF2 and others to predict the prognosis of AML patients [16].

Considering this, we present a set of genetic variants in AML patients receiving NGS in Pakistan in this study Our primary objective was to investigate the frequency of specific genetic variants, percentages of variant allele frequencies (VAF), resultant protein changes and their clinical significance in a series of AML patients. These data hold potential to guide local risk stratification practices and selection of targeted therapy for patients based on their mutational background.

## Methods

This study included nine consecutive adult patients diagnosed with AML via bone marrow biopsy and flow cytometry, who underwent targeted NGS testing for genetic variants presented at Dow University Hospital, Johar campus, Karachi, Pakistan. All patients underwent NGS testing at the time of diagnosis and none had prior exposure to chemotherapy. Consent was obtained from each patient in order to report their genetic/clinical data (anonymized).

### Ethical approval

This study was approved by the Institutional Review Board (IRB) of Dow University of Health Sciences (Ref: IRB-4065/DUHS/EXEMPTION/2025/256), following the 218th IRB meeting held on June 14, 2025. The study was granted exemption status and approval for 1 year. All procedures performed were in accordance with the ethical standards of the institutional research committee.

### Inclusion criteria

Patients included in this study met the following eligibility criteria: a confirmed diagnosis of AML based on bone marrow morphology and flow cytometry; availability of NGS mutation profiling performed at the time of diagnosis; age 18 years or older; are newly diagnosed and have no prior history of chemotherapy at the time of NGS testing.

Due to the high cost of NGS testing in our resource-limited setting, molecular profiling was performed when there was strong clinical justification, typically in younger patients, those with suspected high-risk features or when targeted therapy options were being considered. This selection approach may introduce bias and affect the generalizability of our findings to the broader Pakistani AML population. The NGS panel identified relevant mutations, and these patients were retrospectively followed to assess clinical data, including age, sex, clinical presentation and diagnostic classification.

For each patient, the following data were recorded:

- Demographics (age, sex)
- Molecular mutations detected by NGS, including VAF.
- Identification of MR mutations as per 2022 ICC and ELN guidelines.
- Clinical interpretation regarding prognosis or targetability, based on published evidence and databases such as MyCancerGenome.

### NGS analysis

All NGS testing was handled by BioMarker Solutions Limited, a College of American Pathologists-accredited and International Organization for Standardization (ISO)-certified reference laboratory based in London, UK, using the inDx™ Extended AML/myelodysplastic syndrome (MDS) Panel. The inDx^TM^ Extended AML/MDS Panel contains 29–30 cancer-related genes frequently altered in myeloid malignancies. These genes include both prognostic biomarkers (e.g., *NPM1, DNMT3A, TP53* and* WT1*) and MR genes (e.g., *ASXL1, RUNX1, U2AF1, SRSF2* and* EZH2*), aligned with current diagnostic classifications (ELN 2022, ICC 2022).

Commercially validated kits (either Monarch or Qiagen, depending on the situation) were used to extract genomic DNA from peripheral blood samples (Ethylenediaminetetraacetic Acid). Peripheral blood was used in accordance with the standard protocol of the reference laboratory and was deemed acceptable given the presence of adequate circulating blast counts in AML patients. Libraries were sequenced on Ion Torrent^TM^ platforms (Thermo Fisher Scientific) using proprietary targeted capture protocols. With a minimum of 100–200 reads per amplicon and analytical sensitivity down to <5% VAF, the sequencing coverage satisfied laboratory quality standards. Thermo Fisher Scientific’s Ion Reporter^TM^ bioinformatics software, which makes use of the Torrent Mapping Alignment Program optimized for Ion Torrent sequencing data, was used for sequence alignment and variant calling. Variants were annotated against the human reference genome GRCh37/hg19.

Orthogonal validation using alternative platforms (e.g., Sanger sequencing, allele-specific qPCR or Illumina-based NGS) was not performed, as all reported variants met internal laboratory quality thresholds and were generated using a clinically validated assay operating under external quality assurance programs (EMQN) and ISO-certified laboratory standards (ISO 9001:2015, 15189, 27001). This approach reflects real-world diagnostic practice in resource-limited settings.

Only actionable genetic variants were reported and are discussed herein. These include non-synonymous somatic mutations with confirmed relevance to AML biology or classification as per the latest literature. Copy number variants, fusions and larger chromosomal rearrangements were not assessed by this assay.

## Results

### Patient characteristics

Nine AML patients who underwent NGS were included in this study The median age of the cohort was 42 years (Range: 28–57 years), with a male predominance (7/9, 77.8%).

### Mutation landscape

NGS analysis revealed actionable genetic mutations in 6/9 cases (66.7%). Among these, 5/6 patients had multiple mutations while 1/6 had a single mutation. The remaining 3/9 patients showed no detectable mutations. The total number of mutations found in these 6/9 patients was 13, spanning 11 genes. Detailed case-wise mutation is presented in Table 1.

**Figure 1.**
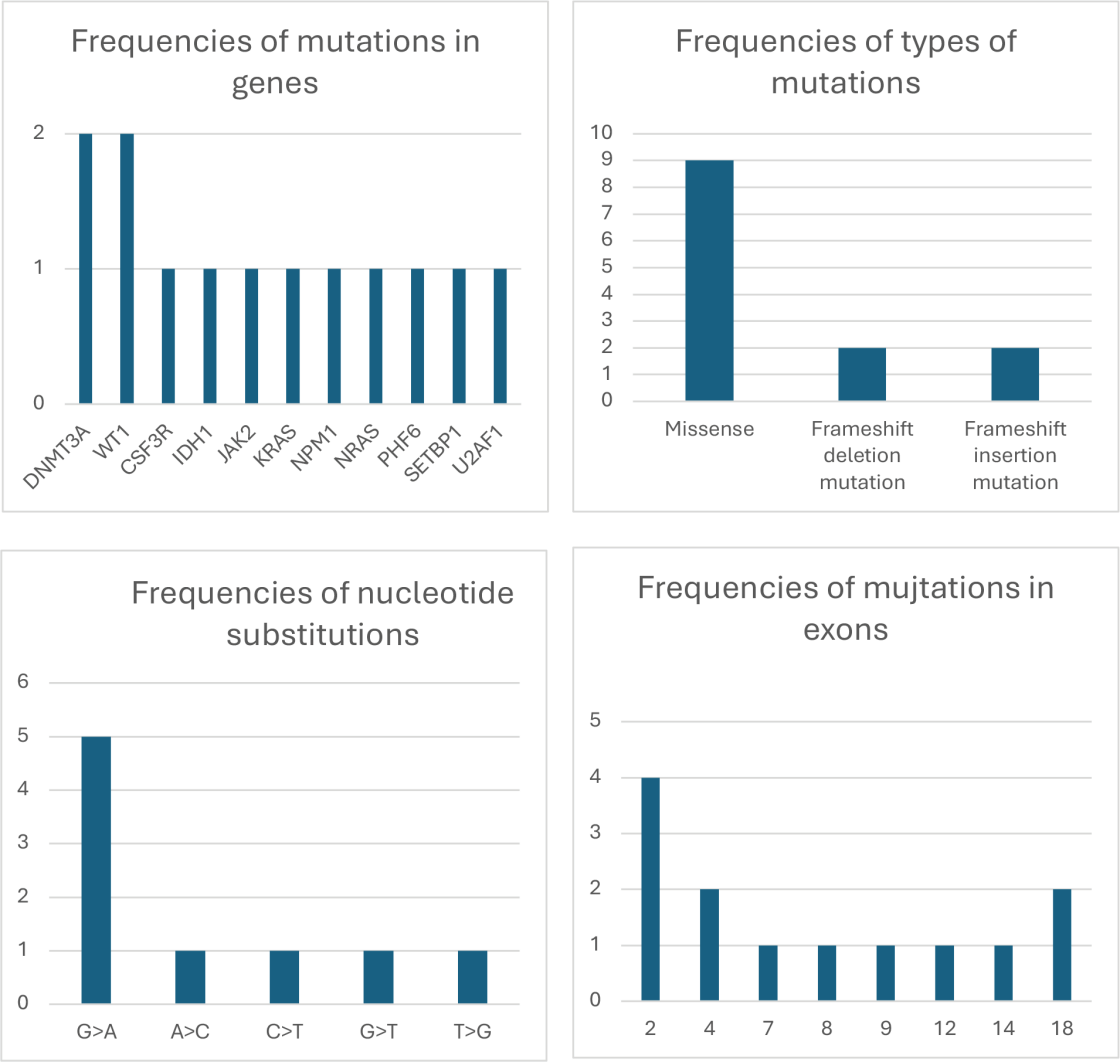
Frequencies of mutations in each gene (a), types of mutation (b), nucleotide substitutions (c) and mutations in exons (d).

Out of these 11 mutated genes, *WT1* and *DNMT3A* were the most predominantly mutated, each affected in two patients (15.4%). Other mutated genes were affected in individual cases (7.7% each), including *CSF3R*, *IDH1*, *JAK2*, *KRAS*, *NRAS*, *PHF6*, *SETBP1*, *U2AF1* and *NPM1*. The distribution of gene frequencies, mutation types, nucleotide substitutions, and exon involvement is illustrated in Figure 1.

**Figure 2.**
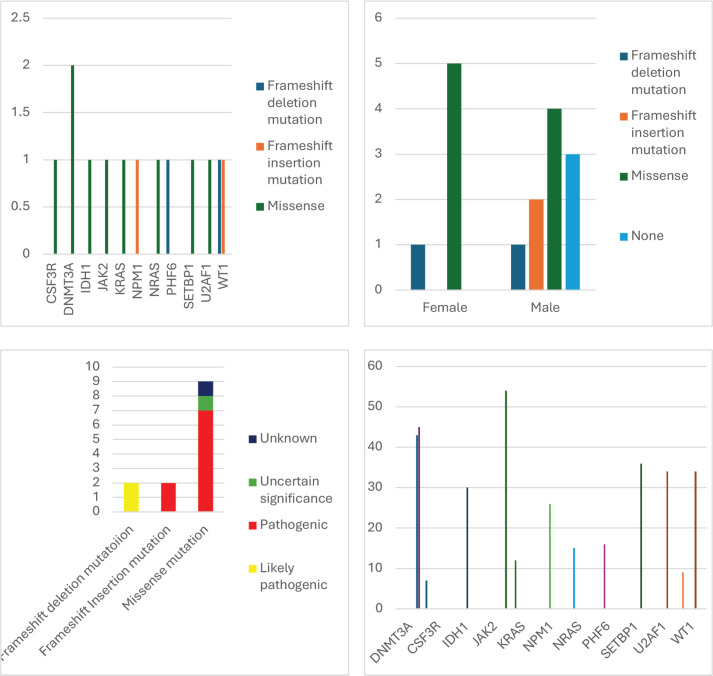
Frequencies of types of mutations (a), across genders (b), across pathogenicity groups (c) and variant allele frequencies of specific mutations (d).

Missense mutations were the predominant type found in 9/13 (69.2%) mutations, while frameshift insertions and deletions were equally represented in 2/13 (15.4% each) of the mutations. Among the nine missense mutations, five involved G>A substitutions. Exon 2 was most frequently affected (4/13, 30.8%), with additional alterations observed in exons 4, 13, 7, 8, 9, 12 and 14. Overall distribution of mutation types, gender-based distribution, pathogenicity categories, and variant allele frequencies is shown in Figure 2.

### Gene-specific findings and mutation patterns

Frameshift deletions were detected in *PHF6* and one of the *WT1* mutations, while *NPM1* and another *WT1* mutation showed frameshift insertions. The rest were missense mutations. In the two female patients, each harbored three mutations, mostly missense (5/6, 83.3%), and one frameshift deletion (1/6, 16.7%). Among males, four of seven had mutations (57.1%), with three showing no variants. A total of seven mutations were seen in the male subset, most were missense (4/7, 57.1%), followed by insertions (2/7, 28.6%) and deletions (1/7, 14.3%).

### Pathogenicity and variant distribution

According to the established classification, 8/13 (61.5%) mutations were pathogenic and 3/13 (23.1%) were likely pathogenic. One mutation each was categorized as of uncertain significance or unknown (7.7% each). All insertion mutations (2/13) were classified as pathogenic, whereas deletions (2/13) were likely pathogenic. Notably, most missense mutations (7/9, 77.7%) were pathogenic. The variant of uncertain significance (VUS) was a WT1 frameshift deletion (c.1311_1312delTT), while the unknown variant was DNMT3A c.1939G>A. These classifications were based on internal bioinformatics pipelines and external variant databases (ClinVar, COSMIC, published literature).

### Co-mutation observations

A subset of cases showed co-mutational patterns: *WT1* occurred alone in one patient and alongside *CSF3R* in another. *DNMT3A* co-occurred with *KRAS* and *NRAS* in one case, and with *NPM1* in another. *IDH1* and *JAK2* were seen together in one patient, while *PHF6*, *SETBP1* and *U2AF1* were simultaneously present in another.

### VAF trends

VAF values ranged from 7% to 54% (Median: 30%). *DNMT3A*, *JAK2*, *SETBP1* and *U2AF1* mutations showed higher VAFs (≥30%), representing dominant clones. In contrast, *WT1*, *CSF3R*, *KRAS* and *NRAS* mutations had lower VAFs (7%–15%). *WT1* mutations showed wide VAF variability (9% versus 34%).

**Figure 3.**
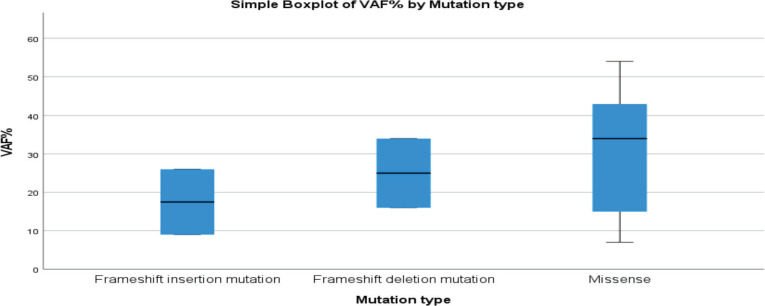
Boxplot of mutation type with VAF%.

No linear correlation between VAF% and patient age was found in the scatterplot. Boxplot comparison by mutation type showed the widest VAF range and the highest median VAF in missense mutations, followed by frameshift deletion and insertion mutations (Figure 3).

### Clinically significant subgroups

**Figure 4.**
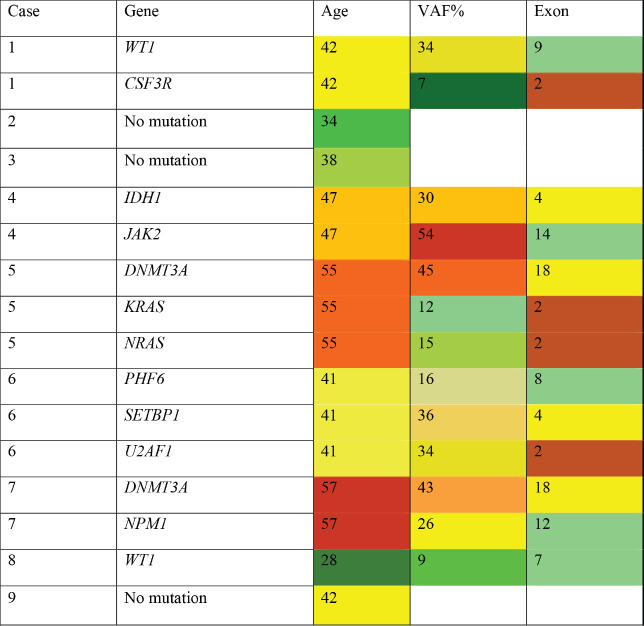
Heat map of age, VAF% and frequencies of exon mutations with genes.

**Figure 5.**
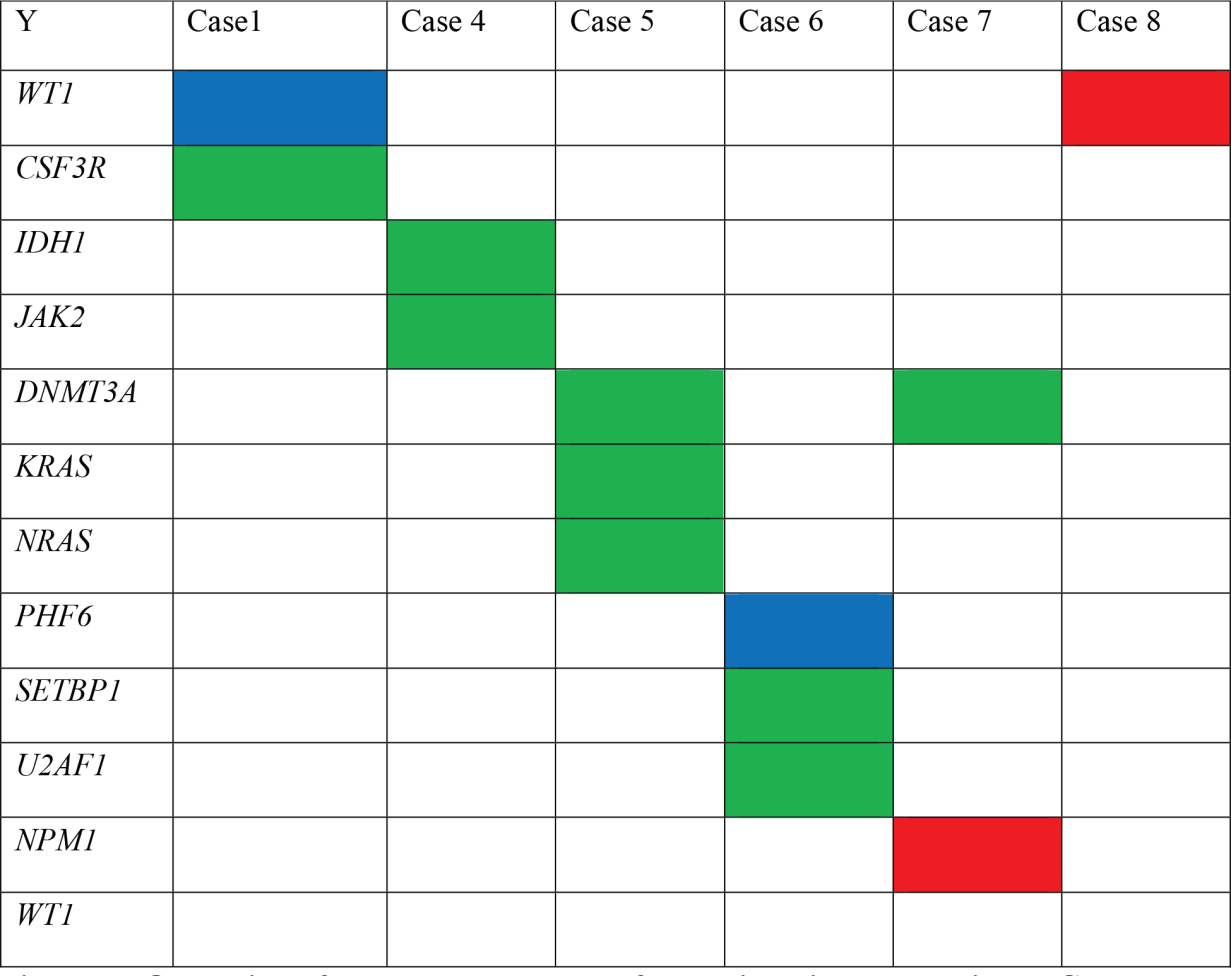
Oncoprint of number and types of mutations in each patient. ^a^Green boxes represent missense mutations, blue represent frameshift deletion and red represent insertion mutations.

- **MR mutation**: One patient had a pathogenic U2AF1 Q157P mutation (VAF %), along with concurrent PHF6 and SETBP1 mutations, consistent with AML-MR as per ELN 2022 and ICC 2022 criteria. This profile suggests a possible transformation from underlying MDS.
- **Favourable risk**: One case carried the *NPM1 W288Cfs*12* mutation (VAF 26%)—a favourable prognostic marker.
- **Targetable lesions**: Dual mutations in *IDH1 R140Q* and *JAK2 V617F* were detected in a single patient (VAFs 30% and 54%, respectively).
- **Heat map analysis:** Integrated visualization of age, VAF%, and exon-level mutation distribution is shown in Figure 4.
- **Oncoprint analysis:** The distribution and co-occurrence of mutations across individual patients is shown in the Oncoprint plot (Figure 5).

Box plot of VAF% by types of mutations revealed a higher median VAF% of missense mutations, followed by frameshift deletions and insertion mutations; however, missense mutations involved a wide variation in VAF%, ranging from 7% to 54%, where both the smallest and the largest VAF percentages were observed for missense mutations. But the sample size was again very small to make a useful interpretation.

## Discussion

This study investigates the mutational landscape of adult AML patients from a Pakistani cohort using targeted NGS. The findings of this study highlight the genetic diversity of AML and emphasize the necessity of incorporating molecular diagnostics in routine clinical workflows, even in low-resource settings.

Several important findings were revealed despite the small sample size. Mutations were observed in 66.7% patients, which is in line with the Kenyan cohort (75%) and the other international datasets [5,17]. Multiple mutations were found in most of the mutated cases, highlighting the complex clonal dynamics of AML. WT1 and DNMT3A were the most frequently mutated genes (15.4% each). The Cancer Genome Atlas data and other literature suggest that WT1 mutations have a poor prognosis and are often found associated with complex or monosomal karyotypes [18,19]. DNMT3A mutations, observed in two patients with high VAF (43% and 45%), suggested early clonal events with clonal dominance. Literature suggests that DNMT3A mutations have a poor prognosis, particularly when co-occurring with FLT3-ITD, despite the fact that FLT3-ITD was not detected in our cohort [20].

Co-occurring IDH1 and JAK2 mutations represented an uncommon but therapeutically relevant combination because specific inhibitors are available for both targets [21]. This finding highlights the need for early implementation of genomic profiling to guide personalized treatment plans.

NPM1, a favourable-risk marker, was co-existing with DNMT3A in one patient. This combination is associated with worse outcomes, aligned with HARMONY Alliance machine learning data, which show that NPM-1 mutations are often associated with three or more concurrent mutations. For instance, the triple mutation pattern of NPM1+FLT3-ITD+DNMT3A predicts poor survival (approximately 33% 2-year overall survival). This highlights the limitations of current ELN 2022 risk stratification models and the need for the development of better predictive models [22].

Co-mutation of DNMT3A with RAS pathway alterations (KRAS/NRAS) was also detected. This suggests convergence of RAS/MAPK pathway activation and epigenetic dysregulation, the two being the critical mechanisms in leukaemogenesis [23]. The co-occurrence of IDH1/JAK2 and NPM1/DNMT3A is consistent with established mutational hierarchies, where NPM1 mutations typically occur after early driver lesions [24].

Low VAFs were observed for WT1, KRAS and NRAS (7%–15%), indicating subclonal evolution, whereas high VAFs (≥30%) were observed for SETBP1, JAK2, DNMT3A and U2AF1, suggesting founder or early clonal mutations [25].

Peripheral blood was considered acceptable because circulating blast counts are adequate in AML, and clonal mutations are typically represented in both compartments, i.e., blood and bone marrow. All samples met the requirements for blast percentage and DNA quality of the reference laboratory. However, future prospective studies would benefit from bone marrow sampling, particularly in cases with low peripheral blast counts or minimal residual disease.

U2AF1 Q157P mutation was found in one patient. It serves as a defining criterion for AML-MR under the ELN 2022 classification [16]. Concurrent SETBP1 and PHF6 mutations indicate disruption of chromatin regulation and RNA splicing machinery, consistent with that observed in Western cohorts [26,27]. This mutational profile supports the hypothesis of transformation from a preleukaemic MDS.

Frameshift mutations were identified in WT1, PHF6 and NPM1. NPM-1 fell into the favourable-risk category according to ELN 2022 guidelines [16]. Three cases in our cohort showed no mutations, which is notable when considering that large-scale studies demonstrate driver mutations in over 95% of AML cases [28]. This discrepancy likely reflects the limited scope of our NGS panel, which may miss structural variants, cryptic fusions or mutations in non-coding regions [29]. This can be addressed by complementary cytogenetics or broader sequencing platforms.

VUS represent a significant challenge in resource-limited settings. A WT1 frameshift deletion classified as a VUS was interpreted as potentially pathogenic. Based on established WT1 biology in AML, it represents a loss-of-function mutation in this tumour suppressor gene. However, the lack of functional validation studies or comprehensive population-specific variant databases hinders definitive clinical interpretation.

One NGS-negative patient was later found BCR-ABL1 positive through polymerase chain reaction. This highlights the limitations of NGS panels, which lack fusion gene detection. This emphasizes the need for integrated molecular testing strategies, particularly in cases of suspected AML or Chronic myeloid leukaemia blast crises.

Missense mutations were the most predominant (69.2%). This was followed by an equal proportion of frameshift deletion and insertion mutations (15.4% each). Most missense and all insertion mutations were classified pathogenic, highlighting their role in leukaemogenesis [30]. Although correlations between VAF and clinical parameters such as age were not statistically significant due to sample size, these findings are vital for initiating large-scale genomic studies in the region.

While morphological and immunophenotypic subclassification data were not systematically available for this retrospective study, correlation of molecular findings with FAB or WHO morphological categories would strengthen future studies. In prospective AML studies, integrating molecular, cytogenetic, morphological and immunophenotypic data with clinical outcomes is advised as standard practice because it offers the most thorough risk stratification.

## Limitations

This study has some important limitations, including a small sample size (*n* = 9), retrospective design and consecutive sampling that limit generalizability. We were unable to validate local outcomes and complete ELN 2022 risk stratification due to a lack of systematic morphological/ immunophenotypic subclassification, thorough clinical outcomes and complete cytogenetic data. For low blast cases, using peripheral blood instead of bone marrow might not be the best option. The targeted NGS panel is unable to identify non-coding mutations, structural variations or fusions (such as BCR-ABL1). No independent validation using orthogonal methods (Sanger sequencing, qPCR or Illumina NGS) was performed. While all variants met the reference laboratory’s quality thresholds under EMQN and ISO standards, independent validation would strengthen confidence in actionable findings. We acknowledge this limitation; however, our primary aim was descriptive, i.e., to demonstrate NGS feasibility and clinical utility in Pakistani AML patients, rather than to establish novel variants or develop new bioinformatics pipelines. Future cooperation with specialized molecular biology labs is necessary to carry out functional studies to confirm pathogenicity, especially for VUS.

## Conclusion

This study shows that targeted NGS has the capability of identifying prognostically significant and clinically actionable mutations in AML, even in low-resource settings. These findings support the wider implementation of molecular diagnostics in routine care and highlight the cruciality of regional mutation mapping in risk assessments and developing treatment plans.

## List of abbreviations

AML, Acute myeloid leukaemia; *BCR-ABL1*, Breakpoint cluster region-abelson murine leukaemia viral oncogene homolog 1; ELN, European Leukaemia Net; EMQN, European molecular genetics quality network; ICC, International Consensus Classification; ISO, International Organization for Standardization; MDS, Myelodysplastic syndromes; MR, Myelodysplasia-Related; NGS, Next-Generation Sequencing; VAF, Variant allele frequency.

## Conflicts of interest

The authors declared no financial or non-financial conflicts of interest.

## Funding

None received.

## Ethical approval

This study was approved by the Institutional Review Board (IRB) of Dow University of Health Sciences (Ref: IRB-4065/DUHS/EXEMPTION/2025/256), following the 218th IRB meeting held on June 14, 2025. The study was granted exemption status. All procedures performed were in accordance with the ethical standards of the institutional research committee and the declaration of Helsinki.

## Figures and Tables

**Table 1.** Case wise details of mutations.

ID	Gene	Mutation	Mutation type	Exon	Protein change	Clinical significance	VAF %
Case 1	*WT1*	c. 1311_1312delTT	Frameshift deletion mutation	9	p. Arg439PhefsTer20	Uncertain significance	34
Case 1	*CSF3R*	c. 244 T>G	Missense	2	p. Tyr814Ter	Likely pathogenic	7
Case 2		None					
Case 3		None					
Case 4	*IDH1*	c. 419 G>A	Missense	4	p. Arg140Gln	Pathogenic	30
Case 4	*JAK2*	c. 1849 G>T	Missense	14	p. Val617Phe	Pathogenic	54
Case 5	*DNMT3A*	c. 1915 C>T	Missense	18	p. Leu639Phe	Pathogenic	45
Case 5	*KRAS*	c. 35 G>A	Missense	2	p. Gly12Asp	Pathogenic	12
Case 5	*NRAS*	c. 38 G>A	Missense	2	p. Gly13Asp	Likely pathogenic	15
Case 6	*PHF6*	c. 834delG	Frameshift deletion mutation	8	p. Met278IlefsTer17	Likely pathogenic	16
Case 6	*SETBP1*	c.2608 G>A	Missense	4	p. Gly870Ser	Pathogenic	36
Case 6	*U2AF1*	c. 470 A>C	Missense	2	p. Gln157Pro	Pathogenic	34
Case 7	*DNMT3A*	c. 1939 G>A	Missense	18	p. Gly646Arg	Unknown	43
Case 7	*NPM1*	c. 863_864insCTTG	Frameshift insertion mutation	12	p. Trp288CysfsTer12	Pathogenic	26
Case 8	*WT1*	c. 898_899insAGACCCG GAACGCCCAGACTA	Frameshift insertion mutation	7	p. Tyr233fsTer	Pathogenic	9
Case 9		None					
